# A link between mitotic defects and mitotic catastrophe: detection and cell fate

**DOI:** 10.1186/s13062-021-00313-7

**Published:** 2021-12-09

**Authors:** Elena V. Sazonova, Svetlana V. Petrichuk, Gelina S. Kopeina, Boris Zhivotovsky

**Affiliations:** 1 Faculty of Medicine, MV Lomonosov Moscow State University, Moscow, Russia 119991; 2 Federal State Autonomous Institution “National Medical Research Center for Children’s Health” of the Ministry of Health of the Russian Federation, Moscow, Russia 119296; 3 Division of Toxicology, Institute of Environmental Medicine, Karolinska Institute, Box 210, 17177 Stockholm, Sweden

**Keywords:** Mitotic catastrophe, Cell death, Senescence, DNA damage, Cancer

## Abstract

Although the phenomenon of mitotic catastrophe was first described more than 80 years ago, only recently has this term been used to explain a mechanism of cell death linked to delayed mitosis. Several mechanisms have been suggested for mitotic catastrophe development and cell fate. Depending on molecular perturbations, mitotic catastrophe can end in three types of cell death, namely apoptosis, necrosis, or autophagy. Moreover, mitotic catastrophe can be associated with different types of cell aging, the development of which negatively affects tumor elimination and, consequently, reduces the therapeutic effect. The effective triggering of mitotic catastrophe in clinical practice requires induction of DNA damage as well as inhibition of the molecular pathways that regulate cell cycle arrest and DNA repair. Here we discuss various methods to detect mitotic catastrophe, the mechanisms of its development, and the attempts to use this phenomenon in cancer treatment.

## Introduction

Back in 1939, Glücksmann and Spear [1] first described a fraction of cells in the mitotic stage that instantly declined in response to radiation and did not reappear until several hours following treatment. Detailed microscopic examination revealed the presence of cells with an abnormal configuration and spatial rearrangements of chromosomes that began to die at or after the first post-radiation mitotic peak (reappearance of mitotic cells) [2]. In 1986, researchers described “mitotic catastrophe” as the lethal phenotype of a yeast strain generated by combining an activated *cdc2* allele with a recessive temperature-sensitive *weel* mutation. This phenomenon was the result of aberrant execution of mitosis—not premature entry into mitosis—with respect to chromosome segregation and septum formation [3]. However, the term mitotic catastrophe to explain the mechanism of cell death linked to delayed mitosis was used for the first time in 1995 [4]. Today, mitotic catastrophe is considered to be an oncosuppressive mechanism that prevents the survival of cells that are unable to complete mitosis due to defects of the mitotic apparatus, DNA damage, and mitotic checkpoint errors [5, 6]. Mitotic catastrophe is not a separate form of programmed cell death (PCD); rather, it is a stage that precedes various types of cell death [7]. Mitotic defects can result from exogenous and endogenous sources. Endogenous sources include mitotic stress caused by the occurrence of aberrant ploidy and high levels of replicative stress [8, 9].

Such disturbances of the mitotic machinery are controlled during mitosis by a special molecular mechanism ensuring correct chromosome segregation, namely spindle-assembly checkpoint (SAC). Inhibition of SAC proteins, which control the assembly of the spindle, causes disruption of chromosome segregation, blocks metaphase, and promotes the development of mitotic catastrophe [10]. This condition may also be a consequence of events occurring in other phases of the cell cycle. Thus, dysfunction of the S-phase checkpoint causes premature transition of cells into mitosis, which can be the root cause of changes that lead to irreversible cell cycle arrest and mitotic catastrophe [11, 12].

**Fig. 1:**
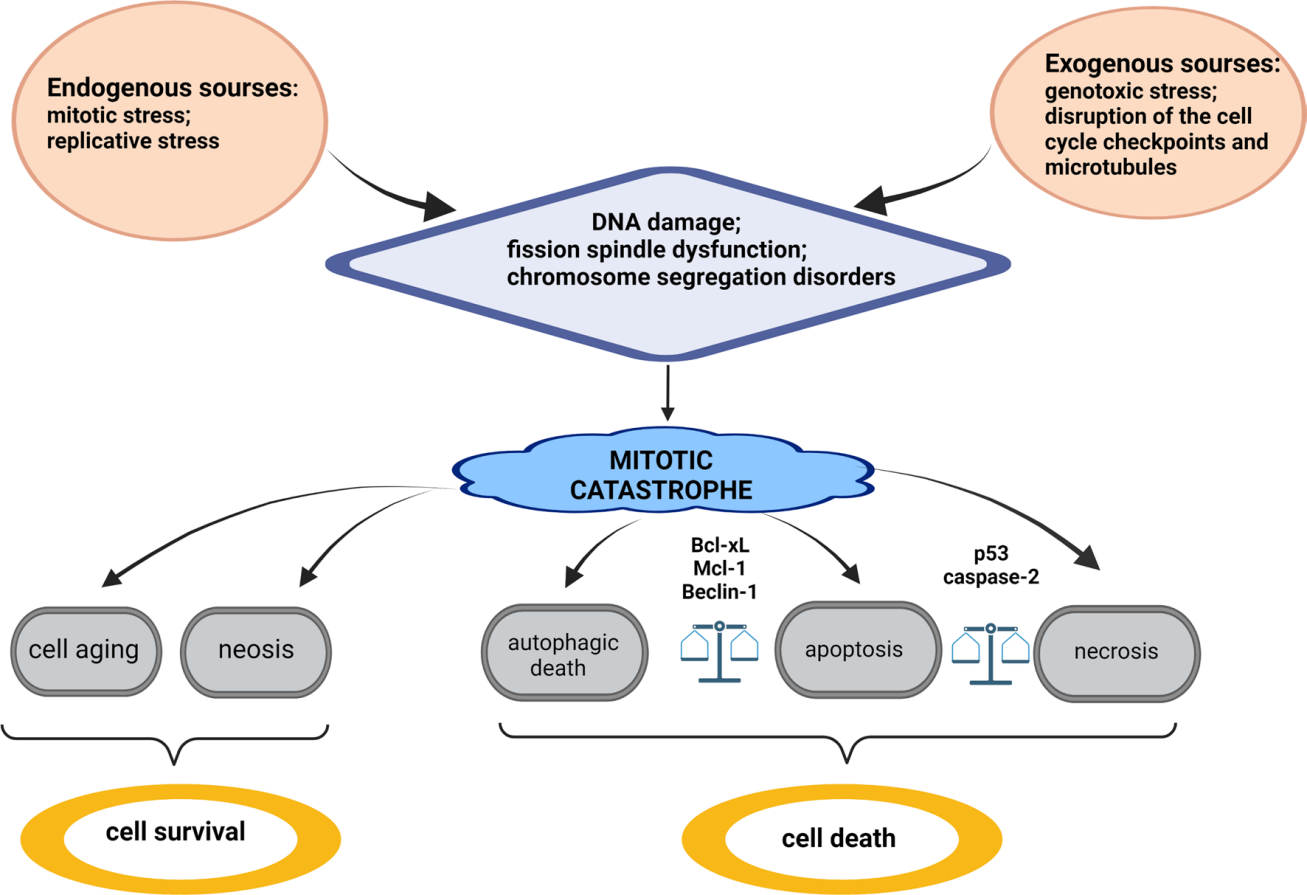
Sources of mitotic catastrophe induction and its outcomes (for details, see the text)

Exogenous sources of various xenobiotics can affect DNA replication, the cell cycle checkpoints, chromosome segregation, microtubule function, and introduce DNA damage [13]. Triggering mitotic catastrophe by exogenous sources is used as a therapeutic approach for cancer treatment. The development of mitotic catastrophe does not always lead to the triggering of PCD and can precede non-lethal processes such as cell aging (senescence) (Fig. 1). Death caused by mitotic catastrophe most often occurs via apoptosis or necrosis. However, it has recently been shown that mitotic catastrophe is also capable of triggering autophagic cell death (Fig. 1) [14].

### Molecular mechanisms of mitotic catastrophe development and termination

The molecular signaling pathways that trigger mitotic catastrophe are still poorly understood. It is known that mitotic catastrophe can be triggered by sublethal DNA damage [15]. Chemotherapeutic genotoxic agents and radiotherapy, acting directly on the cell DNA, introduce primary damage, thus contributing to the development of mitotic catastrophe. Secondary DNA damage occurs with microtubule poisons or SAC and chromosomal passenger complex (CPC) component inhibitors. Disruption of the fission envelope by inhibiting its key components leads to mechanical damage of chromosomes and, consequently, to secondary DNA damage that can also be enhanced by subthreshold caspases and DNase activation.

Several pathways involved in the development of mitotic catastrophe have been described. The question regarding the role of SAC in the development of mitotic catastrophe remains open. There is abundant evidence showing that prolonged SAC activation leads to mitotic catastrophe and subsequent cell death induced by depletion of Aurora A, Ninein, or TOG [6, 16, 17]. On the other hand, it has been shown that in HeLa cells, aphidicolin, a specific inhibitor of DNA polymerase alpha and delta, induces mitosis with damaged DNA and metaphase arrest [18], which forces cells to enter mitotic catastrophe after attempting chromosome segregation. This sequence of events is followed by cell death. Depletion of the SAC proteins Mad2 or BubR1 by small interfering RNA (siRNA) reverses this effect [19]. Moreover, when spindles are not organized correctly and not all kinetochores are attached correctly, SAC is activated and prevents progression through mitosis by inhibiting the anaphase-promoting complex/cyclosome (APC/C) [20]. This in turn leads to stabilization of cyclin B1, prolonged activation of which is associated with mitotic catastrophe [21]. Inhibition of components of CPC, which coordinates the movement of chromosomes and microtubules during mitosis, stimulates the development of mitotic catastrophe [22, 23]. When one of the CPC components, Aurora kinase B (AURKB), is overexpressed, chromosome retardation in metaphase, chromosome segregation errors, and errors in cytokinesis occur, and AURKB inhibition leads to polyploidization and genomic instability [22]. Survivin, an inhibitor of apoptosis protein (IAP), participates in the CPC complex and ensures accurate separation of sister chromatids and microtubule stabilization at the late stages of mitosis. Reduced survivin expression—for example, by specific RNA interference—causes mitosis delay, chromosome displacement, and cell accumulation in prometaphase [23]. Consequently, loss-of-function mutagenesis of the gene encoding survivin can lead to mitotic disturbances. Thus, blocking the CPC complex or altering the levels of proteins regulating its function leads to significant errors in the mitotic process, a phenomenon that stimulates the development of mitotic catastrophe.

In addition, several other proteins regulating mitotic catastrophe have been identified. One of the best known is p53 [14], which influences the choice of the PCD pathway after the development of mitotic catastrophe (Fig. 1) [24]. During genotoxic stress, p53 regulates the cell cycle checkpoint by triggering p21^WAF1/Cip1^ transcription [25]. p21^WAF1/Cip1^ binds to the cyclin-dependent kinases CDK1 and CDK2, inhibiting their kinase activity, which leads to the arrest of proliferation at certain stages of the cell cycle [25]. Such arrest is essential for triggering DNA damage repair. If repair is ineffective or impossible for any reason, or DNA damage is too strong, signaling pathways leading to cell death are initiated. In the case of ineffective cell cycle arrest, the cell continues to divide, accumulating DNA damage, which in turn leads to the development of mitotic catastrophe. This sequence of events is observed predominantly in cells deficient in p53 protein [26]. It is important to note that the main target of p53, which is p21, alters the transcription of Rb in response to cellular stress, not only to inhibit genes required for the cell cycle progression, but also to activate genes implicated in immunosurveillance. p21 sets a time for the repair of stressed cells but induces secretion of bioactive products, including the chemokine CXCL14, that gradually recruit macrophages to the stressed cells. If the damage is repaired, the p21 level normalizes and the cells survive. Otherwise, attracted macrophages trigger the elimination of stressed cells. This mechanism acts as a timer when the immune response transits from surveillance to clearance of stressed cells [27]. Thus, this process might control immunological elimination of cells after mitotic catastrophe.

Another important regulator of mitotic catastrophe is caspase-2, whose activation is closely related to p53 (Fig. 1) [28, 29]. Moreover, caspase-2 participates in the maintenance of genomic stability [30]. The most studied platform for caspase-2 activation is the PIDDosome complex [29], which is related to the function of p53 and p53-induced protein with a death domain (PIDD) [28]. On the other hand, caspase-2 participates in cell cycle regulation by stabilizing p53 via cleaving its inhibitor Mdm2 [31]. This stabilization of p53 and the cleavage of the Bid protein by caspase-2 are essential for the cellular response during abnormal chromosome segregation and mitotic catastrophe development [18]. Polyploid and aneuploid cells are prone to develop mitotic catastrophe. Caspase-2 deficiency causes predisposition of tumor cells to aneuploidy due to dysfunction of the B-cell CLL/lymphoma 9 like (BCL9L) protein [18]. As a part of PIDDosome complex, caspase-2 participates in the elimination of additional centrosomes and regulates ploidy and proliferation of hepatocytes of the regenerating liver, thus allowing cells to avoid entering a mitotic catastrophe state [32, 33]. Moreover, caspase-2 triggers the process of PCD in cells with an abnormal number of chromosomes and centrosomes, also preventing the development of mitotic catastrophe. However, the role of other caspases and proapoptotic proteins in the regulation of these processes remains unclear. For example, there are data about caspase-6-mediated cyclin B1 cleavage [21] and caspase-8-dependent recognition of DNA damage [34], but the importance of these processes for mitotic catastrophe development have not yet been demonstrated. Thus, the molecular mechanisms describing the involvement of caspases in the formation and consequences of mitotic catastrophe require further investigation.

### Characteristics of the mitotic catastrophe phenotype

The development of mitotic catastrophe leads to morphological and biochemical changes in the cell. As described above, aneuploid and polyploid cells are the most susceptible to mitotic catastrophe, as well as cells that have undergone multipolar mitosis [14]. Cells that are unable to complete mitosis have an abnormal increase in the cyclin B1 level and are delayed in the G2/M transition [5, 6, 35], leading to unique morphological nuclear changes, which characterize mitotic catastrophe. Multiple nuclei, macronucleus, and micronucleus are formed as a result of chromosomal dispersal, chromosome breaks, and karyokinesis disorders in metaphase [5, 6].

Under the conditions of mitotic catastrophe, changes occur in the cell nucleus and the mitochondria are also involved. The reconfiguration of the mitochondrial network likewise can be considered a morphological characteristic of mitotic catastrophe. In the state of mitotic catastrophe, cells are characterized by minimal permeabilization of mitochondrial membranes and the release of a small amount of cytochrome *c* from the mitochondria [36]. This phenomenon activates caspases at a level that is insufficient for cell death but suitable to activate DNases, leading to DNA damage and genomic instability [37]. Thus, changes in the mitochondrial network accelerate the development of mitotic catastrophe. It is important to note that induction of mitotic catastrophe by doxorubicin leads to remodeling of the mitochondrial structure and fragmentation of mitochondria in colorectal cancer cells [38]. In addition, in breast cancer cells, mitochondrial fission promotes radiation-induced mitotic catastrophe and increases the cytosolic Ca^2+^ level. A decrease in the levels of the mitochondrial fission proteins dynamin-related protein 1 (Drp1) and fission mitochondrial 1 (Fis1) inhibit the development of mitotic catastrophe but not apoptosis or necrosis [39]. It seems that morphological alterations in the nucleus and mitochondrial network change the mitotic catastrophe phenotype.

The most prominent biochemical characteristic of mitotic catastrophe is mitotic arrest [5, 6]. A characteristic biochemical feature of cells in a mitotic catastrophe state is an abnormal increase in the cyclin B1 level [35]. Interestingly, proteins regulating the cell cycle influence the development of mitotic catastrophe. Thus, inhibition of checkpoint kinase 2 (Chk2) and the 14–3-3σ protein contributes to the development of mitotic catastrophe [40, 41]. The absence of 14–3-3σ protein in colorectal cancer cells stimulates mitotic catastrophe under genotoxic stress; 14–3-3σ knockout cells are unable to maintain the cell cycle arrest and die, passing through the mitotic catastrophe stage [40]. Inhibition of Chk2 in cervical adenocarcinoma cells stabilizes centrosomes and maintains high levels of cyclin B1, causing prolonged activation of CDK1, thereby stimulating the development of mitotic catastrophe. In 14–3-3σ-deficient colorectal cancer cells treated with doxorubicin, Chk2 activation is reduced significantly compared with the wild-type cells, and Chk2 inhibition promotes the induction of mitotic catastrophe in wild-type cells [41]. In glioblastoma cells, inhibition of Wee1 kinase, responsible for CDK1 phosphorylation, promotes checkpoint defects, impairs mitotic entry, and stimulates subsequent mitotic catastrophe [42].

The above-described mechanisms of triggering mitotic catastrophe allow this process to be characterized by the accumulation of a biochemical marker of DNA damage: minor histone H2AX phosphorylated at serine 139 (γH2AX) [24, 37]. Thus, the state of mitotic catastrophe in addition to morphological changes in the cell nucleus and mitochondria is characterized by the cell cycle arrest at the G2/M phase and accumulation of a biochemical marker of DNA damage.

The most significant factor for clinical practice is regulation of the choice of the cell death pathway after mitotic catastrophe [14]. If mitotic catastrophe ends lethally, apoptosis, necrosis, or autophagy will be induced (Fig. 1). This choice is regulated by p53: when absent, the cell preferentially dies by necrosis [24]. Necrotic cell death is associated with proinflammatory effects due to the release of interleukin-1α, which stimulates the proliferation of surrounding cells, promoting tumor tissue growth [43]. If apoptotic cell death is triggered, p53 activates the mitochondrial pathway of apoptosis through transactivation of B-cell lymphoma 2 (Bcl-2) family proteins, distinguished by the presence of a Bcl-2 homology (BH) domain [44]. In response to stimuli, the proapoptotic proteins Bcl-2-associated X protein (Bax) and Bcl-2 homologous antagonist/killer (Bak) are activated and, by oligomerizing, create a channel, providing permeabilization of the outer mitochondrial membrane and release from the intermembrane space into cytoplasm of proapoptotic proteins: cytochrome *c*, second mitochondria-derived activator of caspases/direct IAP binding protein with low pI (SMAC/Diablo), apoptosis-inducing factor (AIF), endonuclease G, and others. The interaction between cytochrome *c* and the cytoplasmic protein apoptotic protease activating factor 1 (Apaf-1) in the presence of dATP/ATP causes oligomerization of the latter, which makes the caspase-recruitment domain (CARD) of Apaf-1 available for binding to initiator procaspase-9. Thus, cytochrome *c*, Apaf-1, and procaspase-9 form an apoptosome protein complex providing activation of caspase-9, which activates the effector caspases, namely caspase-3, -6, and -7 [45]. Moreover, anti-apoptotic proteins myeloid cell leukemia 1 (Mcl-1) and B-cell lymphoma-extra-large (Bcl-xL) of the Bcl-2 family regulate not only permeabilization of external mitochondrial membrane, but also the choice of cell death or survival after mitotic disaster: apoptosis or autophagy (Fig. 1) [46]. In colorectal cancer cells, increased expression of Mcl-1 or Bcl-xL proteins suppresses apoptosis, stimulating autophagy and mitotic catastrophe development [46].

Autophagy is a mechanism of cell survival regulation under various stresses by utilization of cellular components in autophagolysosomes [47]. When mitotic catastrophe is induced by DNA damage, p53 is able to regulate apoptosis and autophagy. Cytoplasmic p53 inhibits autophagosome formation due to FAK family kinase-interacting protein of 200 kDa (FIP200), a component of the autophagic unc-51-like kinase 1 (ULK1) complex [48]. The processes of apoptosis and autophagy interact by means of the anti-apoptotic protein Bcl-2, which regulates autophagy by releasing Beclin-1 from the Bcl-2–Beclin-1 complex when the cell responds to DNA damage, which leads to activation of autophagy [49]. In turn, autophagy can stimulate the development of mitotic catastrophe [46]. Thus, under conditions of mitotic catastrophe at least three types of cell death can be triggered: necrosis, apoptosis, and autophagy, and apoptosis and autophagy are able to regulate each other (Fig. 1).

Mitotic catastrophe is also associated with replicative cell aging, defined as permanent cell cycle arrest in the G1 phase and characterized by telomere shortening. Other types of cell aging, the stress-induced premature senescence (SIPS) and oncogene-induced senescence (OIS), are phenotypically similar to replicative aging but are not associated with telomere shortening and may be coupled with the development of mitotic catastrophe. OIS can be considered a result of the cellular response to genotoxic stress induced by oncogene-mediated DNA damage in normal cells [50]. For example, increased expression of mitotic arrest deficient 2 (Mad2)-interacting protein p31^comet^ causes cellular senescence, which is accompanied by mitotic catastrophe [51]. SIPS is triggered in response to subcytotoxic DNA damage like mitotic catastrophe in proliferating human cells [52]. SIPS and OIS can develop in parallel. However, the development of cell aging under conditions of mitotic catastrophe induction has a negative impact on anticancer therapy. Cells undergoing senescence secrete a large number of compounds that can stimulate cell proliferation. The senescence-associated secretory phenotype (SASP) includes a variety of inflammatory cytokines, growth factors, and proteases that can make the tissue microenvironment favorable for tumor growth [53].

Neosis is a parasexual form of cell division that allow tumor cells to escape senescence [54]. This form of cell division is characterized by the following peculiarities: DNA-damage-induced senescence/mitotic crisis and polyploidization followed by production of aneuploid daughter cells via nuclear budding, and asymmetric cytokinesis and cellularization conferring a limited mitotic life span to the offspring. Neosis can be repeated several times during tumor growth. The neotic progeny, termed Raju cells, has properties of stem cells but they immediately undergo symmetric mitotic division and become mature tumor cells.

SAC proteins are not activated during neosis, leading to errors in chromosome segregation during mitosis and aneuploidy [55]. Unfortunately, it has not been established whether neosis is a mechanism that evades cell death via mitotic catastrophe and whether Raju cells could facilitate tumor self-renewal (Fig. 1) [56].

Thus, mitotic catastrophe can precede two types of cell aging, SIPS and OIS, the development of which negatively affects tumor elimination and, consequently, the therapeutic effect. It has been suggested that giant senescent cells can also undergo neosis, which could be considered a mechanism of recurrent growth of resistant tumor cells after chemotherapy. However, as mentioned above, the triggering of apoptosis or autophagic death positively affects the removal of tumor cells via the step of mitotic catastrophe.

### The role of mitotic catastrophe and DNA repair in anticancer therapy

Because mitotic catastrophe helps to avoid an increase in the number of cells with an abnormal set of chromosomes, this process can be considered an additional mechanism of tumorigenesis suppression. Most tumor cells are aneuploid or polyploid, so they are more susceptible to death due to mitotic anomalies and, therefore, to the induction of mitotic catastrophe [57]. Given that much lower drug concentrations are used to induce mitotic catastrophe, the side effects can be reduced substantially and, therefore, actively utilized in clinical practice [58]. Taxanes are employed to trigger mitotic catastrophe in breast, ovary, and lung cancer, and vinca alkaloids are suitable for the treatment of acute leukemia and Hodgkin’s lymphoma. Drugs of these groups affect polymerization of microtubules, causing mitotic catastrophe.

The use of DNA-damaging agents and radiation exposure in combination with inhibitors of DNA repair stimulates mitotic catastrophe, enhancing the therapeutic effect (Table 1). For example, AZD6738 is an inhibitor of the kinase ataxia telangiectasia and Rad3-related (ATR), which is involved in DNA damage detection and repair initiation in the absence of ataxia telangiectasia mutated (ATM) and p53 also contributes to mitotic catastrophe [59]. Blocking ATR leads to destruction of inhibited replication forks with the formation of breaks in the replicated sister chromatids. In the absence of ATR and p53, double-stranded breaks of two independently generated, partially replicated sister chromatids can be fused by deletions and aberrant chromosomal translocations, leading to mitotic catastrophe and subsequent cell death. Two independent phase 2 clinical trials of the described inhibitor are currently underway (NCT03682289 and NCT04298008). Thus, ATR inhibitors can potentially be used in clinical practice to trigger a mitotic catastrophe.

**Table 1:** Antitumor agents that promote mitotic catastrophe: potential applications and clinical trials

Inhibitor	Mechanism of action	Tumor types for treatment	Potential applications and clinical trials
ATR inhibitor AZD6738	ATR blockade leads to destruction of inhibited replication forks with the formation of breaks in the replicated sister chromatids that can be fused by deletions and aberrant chromosomal translocations	Renal cell carcinoma, urothelial carcinoma, all pancreatic cancers, endometrial cancer	Phase 2 clinical trial: AZD6738 alone and in combination with the PARP-inhibitor olaparib (NCT03682289) [81]
DNA-PK inhibitor NU7026	Increased polyploidy and mitotic catastrophe after low dose irradiation Negative changes in the NHEJ system in a cell-cycle-dependent manner	Cervical cancer	Preclinical study [60]
		Non-small cell lung cancer	Preclinical study [61]
DNA-PK inhibitor M3814	Block repair of radiation-induced double-stranded breaks and promote p53-dependent mitotic catastrophe	Ovarian cancer	Preclinical study [62]
		Fibrosarcoma and lung cancer	Preclinical study [63]
DNA-PK inhibitor AZD7648	This inhibitor in combination with the PARP inhibitor olaparib, doxorubicin, or radiotherapy causes prolonged arrest of the cell cycle in the G2/M transition, accumulation of micronuclei, and chromosomal aberrations that are indicative of mitotic catastrophe	Ovarian cancer	Preclinical study [64]
		HER2 + , HR + and HER2-negative, triple-negative breast cancer	Phase 1–2 clinical trial (NCT03907969) [82]
		HPV-negative head and neck cancer squamous carcinoma	Preclinical study [65]
Mps1/TTK kinase inhibitor CFI-402257	Disrupt chromosome segregation and block metaphase	Advanced/metastatic HER2-negative breast cancer	Phase 1b clinical trial in combination with paclitaxel (NCT03568422) [67]

In addition, more and more data are accumulating concerning the selective inhibitors of DNA-dependent protein kinases (DNA-PKs), which are also involved in the detection of DNA damage and triggering the repair of double-stranded DNA breaks by nonhomologous joining of DNA ends (NHEJ). For example, siRNA-mediated silencing of DNA-PKcs and DNA-PKcs inhibition by NU7026 increases polyploidy and mitotic catastrophe after low-dose irradiation [60, 61]. NU7026 affects the NHEJ system in a cell-cycle-dependent manner in non-small cell lung cancer cells [61]. The efficacy of M3814, an oral DNA-PK inhibitor, in combination with topoisomerase II inhibitors, doxorubicin and etoposide, has been shown in ovarian cancer models [62]. This inhibitor blocks repair of radiation-induced double-stranded breaks and promotes p53-dependent mitotic catastrophe in fibrosarcoma and lung cancer cells [63]. Besides, the selective DNA-PK inhibitor AZD7648 in combination with the poly (ADP-ribose) polymerase (PARP) inhibitor olaparib, doxorubicin, or radiotherapy has been shown to be efficacious in ovarian and breast cancer cell lines [64]. Such combination of drugs causes prolonged arrest of the cell cycle in the G2/M phase, accumulation of micronuclei, and chromosomal aberrations indicative of mitotic catastrophe development. The use of a DNA-PK inhibitor increases the above-mentioned effects as well as decreases survival of tumor cells [64]. Currently, the combination of DNA-PKs and the PARP inhibitor olaparib is in a phase 2 clinical trial (NCT03907969) [65].

As mentioned above, SAC proteins can be involved in the development of mitotic catastrophe. Monopolar spindle 1 (Mps1; also known as TTK protein kinase), a core component of the SAC signaling cascade, is an attractive therapeutic target for cancer treatment. CFI-402257, a selective small-molecule Mps1/TTK kinase inhibitor, shows higher kinase selectivity compared with BAY 1161909, BAY 1217389, or S 81694 which are currently in phase 1 clinical trials (NCT02792465, NCT02138812, NCT02366949, and 2014–002023–10, respectively) [66]. SAC inactivation leading to chromosome missegregation and aneuploidy has been observed in human cancer cell lines treated with CFI-402257. Use of this compound in monotherapy or in combination with an anti-programmed cell death 1 (PD-1) antibody in mouse models of colon cancer inhibits tumor growth at well-tolerated doses [66]. It is important to note that CFI-402257 seems to be promising therapeutic agent for the treatment of advanced/metastatic HER2-negative breast cancer because of its good tolerance. However, it is not clear whether it works better when given with paclitaxel compared with paclitaxel alone [67]. In another study, researchers showed that Mps1 overexpression is a poor prognostic marker for neuroblastoma patients, and incubation of neuroblastoma cell lines with Mps1 inhibitors reversine and Mps-BAY2 leads to polyploidization/aneuploidization of the cells before they undergo mitotic catastrophe [68].

Taken together, the effective triggering of mitotic catastrophe in clinical practice requires not only the induction of DNA damage, but also inhibition of the molecular pathways that regulate cell cycle arrest and DNA repair.

## Methods of mitotic catastrophe detection

There are a number of approaches to detect mitotic catastrophe. As noted above, the state of mitotic catastrophe is characterized by a set of morphological and biochemical changes. Detecting changes in mitochondrial network morphology can be used as an additional parameter. It should be noted that mitotic catastrophe is not equivalent to the number of cells subjected to mitotic arrest. It has been shown that G2/M cell cycle arrest occurs earlier than mitotic catastrophe and affects a much larger population of cells than mitotic catastrophe [46]. The population of cells that are in G2/M arrest has the ability to offset the damage caused by the detrimental stimulus if the stimulus has been eliminated. Such cells are able to return to a normal physiological state after damage repair or continue life with an altered set of chromosomes, chromosomal rearrangements, and genomic DNA mutations. Moreover, if cell death is triggered but stress stimuli are removed, anastasis as a mechanism of cell survival can be activated [69]. Otherwise, if survival is impossible, death pathways are activated in cells that are in mitotic arrest.

There are several qualitative and quantitative methods to detect mitotic catastrophe. Qualitative methods allow researchers to observe the development of this process, and quantitative methods allow researchers to estimate the percentage of the cell population in the state of mitotic catastrophe. The most correct approach for qualitative detection of mitotic catastrophe is the use of microscopic frame-by-frame imaging with high-resolution or real-time imaging. This approach is optimal to determine the moment of its occurrence and is used actively in modern studies [70, 71]. To perform this task, cell boundaries are usually evaluated by bright-field transmission microscopy or live cells are stained with various fluorescent dyes (FITC, Far-RED, 4′,6-diamidino-2-phenylindole [DAPI], Hoechst 33342, etc.) that bind to the cytoplasmic components, organelles (nucleus, mitochondria, lysosomes, and endoplasmic reticulum), or the plasma membrane.

An easy-to-use fluorophore panel is produced by Molecular Probes. For example, CellTracker and BioTracker Cytoplasmic Membrane Dye are used to stain the cytoplasm and plasma membrane, and MitoTraker, LysoTracker, and ERTraker are used to stain mitochondria, lysosomes, and the endoplasmic reticulum, respectively. The cell nucleus is visualized using fluorescent dyes that can penetrate the plasma membrane of a living cell and bind to DNA, such as Hoechst 33342. An important point is that fluorophores with a different excitation and emission profile should be used to stain the cell nucleus and cytoplasm. In addition, microscopic framing or real-time imaging uses cells transfected with plasmids containing genes of the nucleosomal cortex or cytoplasmic components that are cross-linked to fluorescent proteins, such as histone H2B-GFP or actin-GFP [71]. Electron microscopy techniques provide detailed images of the cell nucleus, and marking cell boundaries by staining the cytoplasm or its components allows reliable identification of multinucleated cells, avoiding false detection of a group of cells as a single multinucleated cell [72].

It is essential to detect multipolar mitoses when studying the phenomenon of mitotic catastrophe under conditions of inhibition of spindle component division. Immunofluorescent staining of fixed cells with antibodies against components of the cell division spindle—proteins that make up microtubules, α-tubulin and γ-tubulin—is employed to solve this technical problem by using confocal microscopy. The nucleus is stained with fluorescent dyes that bind to DNA. As noted above, Hoechst 33342 as well as DAPI are the most commonly used dyes. Such staining allows the detection of both normal and multipolar mitoses [73].

**Fig. 2:**
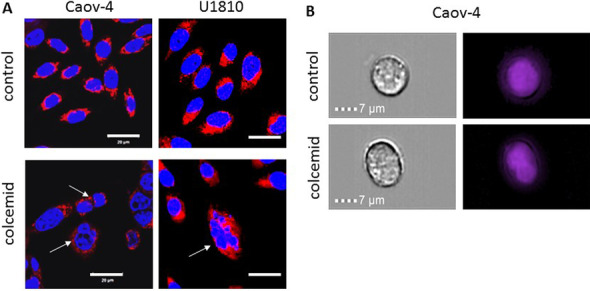
Representative images of morphological changes after induction of mitotic catastrophe were received using classical fluorescent confocal microscopy or imaging flow cytometry. **A** The ovarian carcinoma Caov-4 cells and the lung adenocarcinoma U1810 cells were treated with colcemid 10 ng/mL, a chemotherapeutic agent that depolymerizes microtubules, for 48 h. Cells were grown, treated, fixed in 4% paraformaldehyde, and stained with MitoTracker Red FM and DAPI directly on coverslips. Mitotic catastrophe development was evaluated by analysis of nuclear morphology using a 63 × /1.4 oil objective lens of a LSM 780 confocal laser scanning microscope (Zeiss). After the colcemid treatment of the cells the multi- and micronucleation were detected. **B** The ovarian carcinoma Caov-4 cells were treated with colcemid 10 ng/mL for 48 h. After incubation, the cells were collected by trypsin–EDTA and transferred to conditioned medium. The cells were centrifuged and washed twice with cold phosphate-buffered saline. Then, the cells (2.0 × 10^5^) were resuspended in PBS and DAPI was added to the sample and incubated in the dark at 4 °C for 15 min. After incubation, the cells were analyzed by imaging flow cytometry with the ImageStream Mark II - AMNIS (Millipore Merck, Germany). After the colcemid treatment of Caov-4 cells the multinucleation was detected

For quantitative evaluation of mitotic catastrophe, it is recommended to analyze the nuclear morphology of at least 300 cells in several independent experiments to assess its development reliably. Confocal fluorescence microscopy techniques are used to detect the nuclei, stained with Hoechst 33342 or DAPI; fixed cell cytoplasm components, stained with CellBrite, LysoTracker, MitoTraker, or ERTraker; and/or spindle components such as tubulin. This approach to analyze mitotic catastrophe is utilized in many laboratories [74–76]. Thus, all forms of nuclear morphology changes, including multinucleation, micronucleation, and multipolar mitosis, are detected reliably by using this approach. For example, we identified these changes in the ovarian carcinoma cell line Caov-4 and the lung adenocarcinoma cell line U1810 after treatment with colcemid, which depolymerizes microtubules (Fig. 2A; for technical details, see the figure legend).

The development of new technologies has permitted the use of flow cytometry associated with imaging the nucleus to assess macronucleation and multinucleation during mitotic catastrophe. Application of this quantitative approach makes it possible to obtain an image of the nucleus with the cell image superimposed on it. The nucleus is stained with propidium iodide or another fluorophore that binds to DNA, and the cell morphology is detected by imaging flow cytometry [77]. For example, we detected the population of multinucleated Caov-4 cells after colcemid administration (Fig. 2B; for technical details, see the figure legend). Unfortunately, analysis of the nuclear morphology of cells by the above-mentioned technique is not able to detect micronucleation accurately due to insufficient resolution of this approach. Flow cytometry records the optical parameters of cells or particles in the flow by light scattering and fluorescence signals in a piece-by-piece analysis mode [78]. To focus the cells in the liquid stream, hydrodynamic or acoustic focusing is used to line up the cells in the stream, one by one. Cells are irradiated with a laser, the optics of the cytometer collect the light signal from the cells, and electronics convert and digitize the signal for further analysis. The imaging function gives the possibility to analyze the cellular morphology: for example, to distinguish necrotic and apoptotic morphology [77]. However, changes such as micronucleation could be lost during calculation (unpublished data). Taken together, imaging flow cytometry may help to count cells with nuclear changes specific for mitotic catastrophe. However, this method is only useful to detect macronucleation and multinucleation and not micronucleation.

Confocal fluorescence microscopy as well as a wide range of fluorophores allow researchers to obtain clear images of different biological structures in different scanning planes and to detect micronucleation [79, 80]. Thus, researchers have recommended various imaging approaches to detect mitotic catastrophe; however, confocal fluorescence microscopy is recommended to obtain convincing results.

## Conclusion

Mitotic catastrophe is an irreversible arrest of the cell cycle that develops when severe chromosomal aberrations or dysfunctions of the mitotic spindle occur, causing cells to fail to complete mitosis. Depending on the balance between certain proteins in the cell, mitotic catastrophe can end in a lethal process, such as apoptosis, autophagic cell death, or necrosis, or a cell survival program can be triggered by the development of cellular senescence. Despite considerable interest from researchers regarding this process, the mechanisms of mitotic catastrophe development remain insufficiently studied.

The use of DNA damaging agents and radiation exposure in combination with inhibitors of DNA repair systems stimulates mitotic catastrophe, enhancing the therapeutic effect. Several inhibitors of the molecular pathways that regulate the cell cycle arrest and DNA repair have been studied actively as mitotic catastrophe inductors that could be used in cancer therapy. Detection of mitotic catastrophe involves painstaking work to assess cellular morphology and biochemical parameters. Thus, despite the technical difficulties in studying the phenomenon of mitotic catastrophe, its role in the pathogenesis of various types of cancers makes it important to study further.

## Data Availability

Not available.
